# Draft Genome Sequence of NDM-1-Producing *Klebsiella pneumoniae* Clinical Isolate 303K

**DOI:** 10.1128/genomeA.01069-13

**Published:** 2013-12-19

**Authors:** Yu-Chieh Liao, Ying-Hsiang Chen, Hsin-Hung Lin, Jung-Jung Mu, Ho-Sheng Wu, Feng-Chi Chen, Chao Agnes Hsiung

**Affiliations:** Division of Biostatistics and Bioinformatics, Institute of Population Health Sciences, National Health Research Institutes, Miaoli, Taiwana; Center for Research, Diagnostics and Vaccine Development, Centers for Disease Control, Taipei, Taiwanb

## Abstract

Multidrug-resistant New Delhi metallo-β-lactamase 1 (NDM-1)-producing bacteria have spread globally and become a major clinical and public health threat. We report here the draft genome sequence of the *Klebsiella pneumoniae* clinical isolate 303K, harboring an NDM-1 coding sequence.

## GENOME ANNOUNCEMENT

The emergence and spread of carbapenem-hydrolyzing β-lactamases (carbapenemases) over the decades is a point of concern. New Delhi metallo-β-lactamase 1 (NDM-1) is the most recently identified carbapenemase and is now disseminated worldwide. The first detection of NDM-1-producing *Enterobacteriaceae* in Taiwan was in a sample derived from a Taiwanese man who had been through a surgical procedure in India. The patient did not present with symptoms of infection after returning to Taiwan. *Klebsiella pneumoniae* 303K was isolated from a rectal swab culture obtained from this patient (1).

The genomic DNA of isolate 303K was obtained using the DNeasy blood and tissue kit (Qiagen), according to the manufacturer’s recommendations, and was sequenced using a HiSeq 2000 platform (Illumina, USA). A total of 22,428,842 paired-end reads of 101 bp in length, with an average insert size of 329 bp, were generated by a commercial sequencing service provider (Yourgene, Taiwan). Five different genome assemblies generated separately using ABySS 1.3.4 (2), Edena version 3.130110 (3), SOAP*denovo* 2.04 (4), SPAdes 2.5.0 (5), and Velvet 1.2.09 (6) were subsequently integrated into an assembly using CISA (7). This assembly was then integrated with the continuous long-read sequences generated by the SMRT technology (with a 10-kb library) (Pacific Biosciences) to yield the draft genome sequence. The draft genome sequence of 303K consists of 52 contigs, with a G+C content of 57.2%, for a total length of 5,650,292 bp. The assembly was annotated using the NCBI Prokaryotic Genomes Automatic Annotation Pipeline (PGAAP), resulting in the identification of 5,274 coding genes and 79 tRNA genes. Multilocus sequence typing (MLST) analysis was performed on the MLST online server, which assigned sequence type 15 (ST15) to this isolate (8).

The draft genome sequence was analyzed by ResFinder for the identification of drug resistance genes (9). The genes predicted to convey antibiotic resistance to this isolate include *aadA1*, *aph*(*3′*)*-Ia*, *bla*_NDM-1_, *bla*_SHV-12_, *bla*_TEM-1_, *qnrB1*, *mph*(A), and *arr-2*.This finding is consistent with previously reported experimental results (1).

### Nucleotide sequence accession numbers.

This whole-genome shotgun project has been deposited in GenBank under the accession no. AVAN00000000. Described in this paper is the first version, AVAN01000000.
